# Liebig in Great Britain

**Published:** 1845-08-01

**Authors:** 


*Liebig in Great Britain.*—The distinguished Chemist, Liebig, has lately visited England and Scotland, where he has been greeted with much enthusiasm. At Glasgow he received the compliment of a public dinner, which was honored by the presence of titled dignitaries, and distinguished savans. The speeches, &c. are reported in a late number of the London Lancet. This periodical, by the way, has published a course of lectures on Chemistry, by Liebig, corrected by himself, which must possess great interest and value.

